# Emergence and Characterization of a Novel Reassortant Canine Influenza Virus Isolated from Cats

**DOI:** 10.3390/pathogens10101320

**Published:** 2021-10-14

**Authors:** Jin Zhao, Wanting He, Meng Lu, Haijian He, Alexander Lai

**Affiliations:** 1 College of Veterinary Medicine, Nanjing Agricultural University, Nanjing 210095, China; vet_he@163.com (W.H.); lm98981128@163.com (M.L.); 2 Agricultural College, Jinhua Polytechnic, Jinhua 321007, China; 3 School of STEM, Kentucky State University, Frankfort, KY 40601, USA

**Keywords:** novel influenza virus, cats, reassortant

## Abstract

Cats are susceptible to a wide range of influenza A viruses (IAV). Furthermore, cats can serve as an intermediate host, and transfer avian influenza virus (AIV) H7N2 to a veterinarian. In this report, a novel reassortant influenza virus, designated A/feline/Jiangsu/HWT/2017 (H3N2), and abbreviated as FIV-HWT-2017, was isolated from nasal swab of a symptomatic cat in Jiangsu province, China. Sequence analysis indicated that, whilst the other seven genes were most similar to the avian-origin canine influenza viruses (CIV H3N2) isolated in China, the NS gene was more closely related to the circulating human influenza virus (H3N2) in the region. Therefore, FIV-HWT-2017 is a reassortant virus. In addition, some mutations were identified, and they were similar to a distinctive CIV H3N2 clade. Whether these cats were infected with the reassortant virus was unknown, however, this random isolation of a reassortant virus indicated that domestic or stray cats were “mixing vessel” for IAV cannot be ruled out. An enhanced surveillance for novel influenza virus should include pet and stray cats.

## 1. Introduction

Influenza A virus infects a wide range of host species, from birds to mammals, and it exhibits varying degrees of host adaptation [1]. There has been recent increases in cross-species transmission of H3-subtype avian-origin influenza virus (AIV) to mammalian species, including pigs, cats, and dogs [2,3,4]. Cats have recently become a noticeable host for influenza infections [5,6,7]. In December 2016, a low pathogenic avian influenza (LPAI) A (H7N2) virus was identified as the causative source of an outbreak of respiratory illness in a cat shelter, New York. A veterinarian was infected, possibly a result of occupational exposure to infected cats. This report highlighted the threat of public health risk by cats infected with influenza viruses [8]. Of note, after the H3N2-subtype canine influenza virus (CIV) emerged in South Asia, CIV H3N2 infections in domestic cats were reported soon after, indicating the susceptibility of and the risk associated with this host species in the emergence of novel influenza virus [4]. Experimentally infected cats indicated that felines are aberrant hosts (as are humans and other mammals), with symptoms and characteristic lung lesions, and by its limited spreading [9,10].

Interspecies transmission of CIV H3N2 to domestic cats in South Korea [4] highlighted a significant public health risk, as pet cats and dogs come into very contact with humans. Both cats and dogs have alpha-2,6 and alpha-2,3 receptors in the trachea and lung, they can serve as intermediate hosts for avian- and mammalian-origin influenza viruses [11,12]. Furthermore, because of their carnivorous behavior, particularly towards birds, cats are at high risk for AIV infection. These infections provide a condition for mammalian adaptation for further interspecies transmission to other mammalian species, including humans. Despite the fact that cats had been shown to be susceptible to CIV H3N2 [13] by experimental transmission, the situation in domestic cats was unknown, particularly in China where the pet cat population has exploded in recent years.

In this report, we characterized a novel reassortant feline influenza H3N2 virus which was randomly isolated from a symptomatic cat. Diagnostic mutations—which were associated with mammalian adaptation—were found. The current COVID-19 pandemic illustrates the importance of the surveillance of novel respiratory viruses in humans, cats and other pet animals. Enhanced surveillance should be instituted to prevent emergence of novel influenza virus or SARS-CoV-2-like viruses to reduce this public health risk.

## 2. Material and Method

### 2.1. Virus Isolation

The nasal swab was obtained from a four-month-old female British Shorthair cat treated by a veterinary clinic in Nanjing, Jiangsu province of China, in March 2017. The cat exhibited fever, cough, and loss of appetite. After treatment with antibiotics and interferon-α for two weeks, the cat fully recovered. A follow-up investigation found that the cat often ventured out and had frequent contact with other stray cats or dogs. Polymerase chain reaction (PCR) was performed on the sample to detect common respiratory infections in cats for feline calicivirus (FCV), feline herpesvirus type 1 (FHV-1) and feline panleucopenia virus (FPV), using specific primers [14,15,16,17,18]. To isolate the influenza A virus, the nasal swab was inoculated into 10-day-old specific pathogen-free (SPF) embryonated chicken eggs. The allantoic fluids were collected at 48 h post-inoculation, and the hemagglutination (HA) test was performed using chicken erythrocytes (RBCs) [2]. The isolate was identified via a hemagglutination inhibition (HI) test [19] with the reference positive chicken sera according to procedures recommended by the World Organization for Animal Health (OIE). At the same time, serum samples from early-stage and convalescence of the index cat were tested. Briefly, 25 μL of serial 2-fold dilutions of the serum were mixed with four hemagglutinin units (HAU) of virus tested in V-shaped microtiter plates and kept for 30 min at room temperature. Then, 50 μL of 1.0% chicken RBCs were added to each well and incubated at room temperature (22 °C to 25 °C) for 30 min. The HI titer was expressed as the reciprocal of the highest serum dilution that completely inhibited the hemagglutination of four HAU of virus.

### 2.2. RT-PCR, Sequencing and Analysis of Sequences

Viral RNA was extracted using Trizol reagent (Invitrogen, Carlsbad, CA, USA). RNA concentrations were measured by a spectrophotometer (260 nm/280 nm), followed by generation of cDNA with Reverse-transcription Kit (TaKaRa, Dalian, China) using a universal 12bp primers (AGCAAAAGCAGG). PCR amplification was performed using universal primers for influenza A virus [20]. PCR products were cloned into pMD-18T (TaKaRa) and sequenced. The Seqman program was used to compile and analyze the sequences. The nucleotide sequences of the isolate and the reference viruses were added into the MegAlign program and conducted the ClustalW analysis to generate the homology between the isolate and the reference viruses. Maximum-likelihood (ML) phylogenetic trees were generated using the IQ-TREE v 2.1.2 with automatic model selection functionality to select most suitable nucleotide substitution model [21,22]. The bootstrap values were set at 1000 bootstrap replicates. The sequences of the virus were submitted to GenBank (Accession numbers: OK357996-OK358003)

## 3. Results

### 3.1. Virus Isolation

The nasal swab did not cause cytopathic effects (CPE) until the fourth passage on CRFK cells, and PCR results were negative for FCV, FHV-1 and FPV (data not shown). After the nasal swab was inoculated into SPF embryonated chicken eggs for 48 h, the allantoic fluids were assayed by agglutination of chicken RBCs in a HA text. Additionally, hemagglutination-inhibition (HAI) assay with specific antisera determined that it was a H3 subtype influenza virus. This virus was designated as A/feline/Jiangsu/HWT/2017 (H3N2) (FIV-HWT), and it was selected for further characterization. The HI test of the sera in early-stage and convalescence of the index cats were positive against H3N2 FIV-HWT, hence establishing the etiology.

### 3.2. Nucleotide Sequencing and Phylogenetic Analysis

A total of eight gene segments from strain FIV-HWT were amplified by RT-PCR using the universal primers for influenza A virus, followed by cloning into pMD-18T for sequencing. 

The homologous sequences for each segment were aligned by basic alignment search tool (BLAST, http://blast.ncbi.nlm.nih.gov/Blast.cgi. (accessed on 11 September 2021, for verification)). Analysis showed that all of the gene segments, except NS, displayed a close relationship to CIV H3N2 from China, and the nucleotide sequence identity ranged from 98.1–99.8%, as shown in Table 1. The NS gene was highly related to that of human influenza A virus, A/Victoria/55/2015(H3N2), with a similarity of 99.42% (Table 1). The highest nucleotide sequence similarities between the isolate and the FIVs from Korea was 97.8% for the HA, 97.2% for the NA, 97.2% for the PB1, 97.8% for the PB2, 98.3% for the PA, 99.5% for the NP, 98.5% for the M and 97.4% for the NS. 

**Figure 1:**
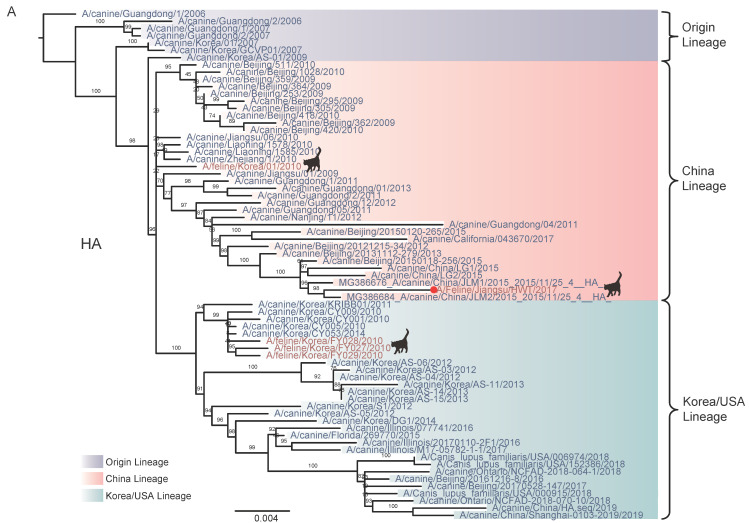

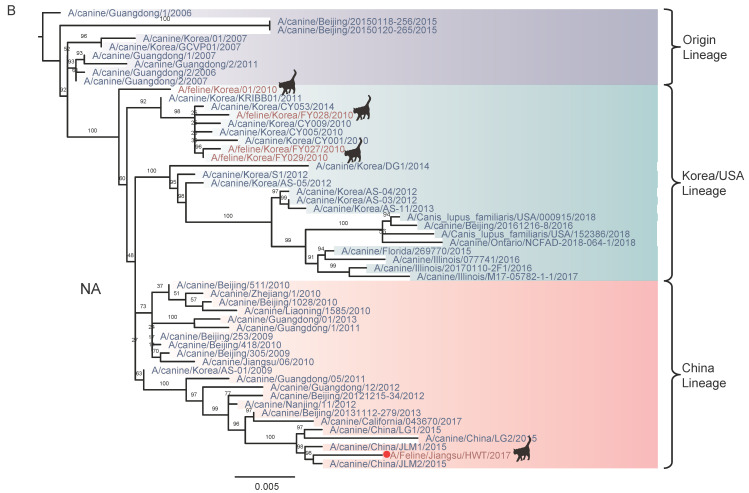
Phylogenetic analysis for the HA and NA gene of the H3N2 influenza viruses. The trees were generated by the maximum-likelihood (ML) with a bootstrap value of 1000 replicates (shown for each node). Viruses isolated in the present study are labeled with red dot, and other isolates from cats added the silhouette of the cat. Different color blocks represent different lineage. (**A**) Phylogenetic tree for HA. (**B**) Phylogenetic tree for NA. Scale Bar: Nucleotide substitutions per site.

**Figure 2:**
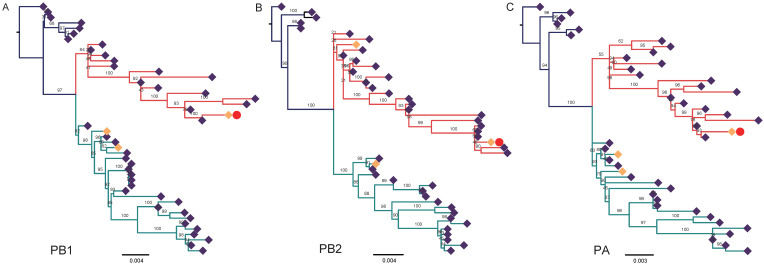

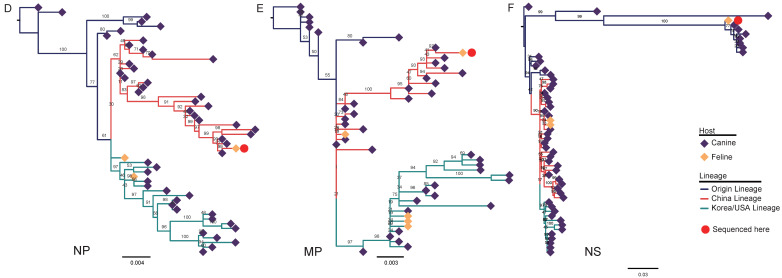
Phylogenetic trees of the internal gene segments of the H3N2 influenza viruses. The trees were created by the maxi-mum-likelihood (ML) with a bootstrap value of 1000 replicates (shown for each node). Viruses isolated in the present study are labeled with red dot and other isolates from cats are labeled with yellow rhombus. The isolates from canine are labeled with blue violet rhombus. Branches of different colors represent different lineages. (**A**–**F**) Phylogenetic tree for PB1, PB2, PA, NP, MP, and NS, respectively. Scale Bar: Nucleotide substitutions per site.

Phylogenetic analysis revealed that the eight gene segments were clustered very closely to those of CIV H3N2 circulating in Guangdong, Jiangsu, and Heilongjiang, between 2006 and 2013 (Figure 1 and Figure 2). The HA (Figure 1A) and NA (Figure 1B) were closely related to those of A/canine/Nanjing/11/2012 (H3N2), and the PB2 (Figure 2A), PB1 (Figure 2B), PA (Figure 2C) were also closely to the Guangdong CIV H3N2. However, the NS (Figure 2F) was more closely related to human virus A/Victoria/55/2015 (H3N2). The phylogenetic results demonstrated that FIV-HWT was probably originated from CIV H3N2, but as a reassortant with the NS from human influenza virus H3N2.

Viruses isolated in the present study has the cleavage site for HA2 to be PERQTRGLL, with a single 332L compared to 332F for the reference sequences (Table 2). This amino acid sequence is characteristic of LPAI. For the HA, with Q226 and G228 at its receptor binding sites (Table 2), suggesting that this virus preferentially binds to avian-like NeuAca2, 3-Gal receptors [23]. Of note, the antigenic sites are conserved among the CIVs (data not shown). Comparing to the deduced amino acid sequences of the NA protein of the FIVs, an inserting of three amino acids (KEI) was detected at position 75–77 (Table 2). The effect of this insertion is unclear. For drug-resistance, diagnostic amino acid residues such as 119E, 222I, 274H, and 292R were present in the NA (Table 2), suggesting sensitivity to neuraminidase inhibitors. The amino acid residues from other FIVs and CIVs at positions 26, 27, 30 and 31 in the M2 protein were L, V, A, and S, respectively. To date, for the influenza viruses examined, 31N was only present in A/feline/Guangdong/1/2012 (H3N2) (Table 2), suggesting a reduction in the sensitivity to amantadine and rimantadine [24]. However, A31I was not found in any isolates from China. In addition, FIV-HWT-2017 has the conserved 627E and 701D in PB2 (Table 2), suggesting a low virulence for mice and humans.

## 4. Discussion

It has been documented that several RNA viruses had cross-species as “spillover” from domestic and wild animals to humans through cross-species transmission, resulting in the most notorious emerging zoonotic diseases, as represented by SARS-CoV-2 and influenza viruses [25,26,27]. Furthermore, the ability of rapid evolution with frequent recombination renders RNA viruses a significant public health risk [28,29]. The identification of a CIV H3N2 infection in cats was unsurprising, as a 2005 report describing tigers in Thailand fed with poultry contaminated by H5N1 became sick and died of the infection [30]. Reports of infection in cats by various subtypes of avian and mammalian influenza virus (AIV H5N1, H5N6, H7N2, pandemic H1N1, and CIV H3N2) have recently increased [8,31,32,33,34,35]. Furthermore, since both α-2,3- and α-2,6-sialic acid-receptors are present in the epithelial cells at the respiratory tracts and gastrointestinal tracts, cats are susceptible to natural and experimental influenza virus infections [4]. However, most of above reports of interspecies infections were sporadic. Possibly, these viruses were not able to sustain in cats because of inefficient transmission.

The situation regarding CIV H3N2 infection in domestic and stray cats remained unknown. In this study, we had isolated and characterized a novel reassortant feline influenza virus from CIV H3N2 from a symptomatic pet cat. Phylogenetic and molecular characteristics of this isolate had shown that this virus is closely related to CIV H3N2 circulating in China, but the NS1 gene was from human influenza A virus, most closely related to A/Victoria/55/2015 (H3N2). There was no more than 3% divergence in the nucleotide and the amino acid sequences among the HA and NA genes of all tested strains. Of note, a CIV H3N2 isolate from a cat had several non-synonymous substitutions to that of CIV H3N2 [4], but there were no more than 2% divergence in its genes. These results may suggest that these sequences were highly conserved, probably a result of antigenic pressure, despite the host range shift by this interspecies transmission. 

The ML tree (Figure 1 and Figure 2) also showed that these seven viral genes (PB2, PB1, PA, HA, NP, NA, M) isolated from this symptomatic pet cat were almost identical to those of the canine influenza H3N2 virus. In the previous study, the G186V, G225D and Q226L/I substitutions of the HA gene were the major contributors to the high-affinity binding of this virus to human receptors, which preferentially bind to α2,6-linked sialic acids (SA). This shift in receptor binding increases the likelihood of upper-respiratory-tract transmission [9]. A signifier of highly pathogenic avian influenza virus (HPAI) is a polybasic (basic amino acid residues RKRT) cleavage site in the HA protein [36]. This site, along with amino acid sequence analysis of the PB2, HA, NA, and M gene of CIV H3N2 isolated from dogs and cats, and from this reassortant virus, were not found. Furthermore, given the evidence of transmission between infected cats and humans [8], this public health risk should not be overlooked.

Of note, despite the limitation of current report that only one isolate was identified so far, the fact that the human NS gene was included in this reassortant is significant. The NS gene had been shown to play a significant role in the virulence [37,38] by modulating cytokines [39], specifically as an antagonist to alpha-interferon. Whether this novel virus, FIV-HWT-2017, has increased virulence by interfering the host immune response remains to be determined. Furthermore, the number of pet cats in this region significantly increased in the past decades. Moreover, as companion animals, cats have a special status in modern human life. The high frequency of close interactions between cats and dogs presents a high for multiple cross-species virus transmission.

Previous reports had indicated that CIV H3N2 had established as an enzootic infection in dogs from eastern provinces of China. With this host species expanding into cats, considering that China had been known as an “epicenter” for the emergence of novel influenza viruses, enhanced surveillance is warranted. We are currently conducting serological survey to evaluate the extent of CIV H3N2 as well as FIV-HWT infection in dogs and cats from this region. Furthermore, pets had been reported infected with SARS-CoV-2, due to the close and frequent contact with humans, their potential role in influenza virus and SARS-CoV-2 should be further investigated. 

## 5. Conclusions

We have shown molecular evidence for a reassortant feline influenza virus derived from CIV H3N2 and from human H3N2. Given the proximity to humans as pets, and their unique behavior towards birds, cats may play a more significant role in the ecology of influenza virus, and as potential mixing vehicle for interspecies transmission and the generation of novel influenza virus with pandemic potential.

## Figures and Tables

**Table 1:** Influenza A viruses with the highest nucleotide sequence identity to the HWT isolate.

Gene	Nucleotide Sequence Compared	Identity (%)	Virus Designation	GenBank Accession Number
PB2	2280	98.6	A/canine/Guangdong/12/2012 (H3N2)	KF826944
PB1	2274	98.1	A/canine/Guangdong/12/2012 (H3N2)	KF826945
PA	2151	98.7	A/canine/Guangdong/12/2012 (H3N2) A/canine/Heilongjiang/L1/2013(H3N2)	KF826946 KF042275
HA	1701	98.2	A/canine/Nanjing/11/2012 (H3N2)	KF322105
NP	1497	99.8	A/canine/Guangdong/05/2011 (H3N2)	JX414247
NA	1417	98.7	A/canine/Nanjing/11/2012(H3N2)	KF322106
M	982	99.0	A/canine/Guangdong/12/2012(H3N2)	KF826950
NS	838	99.42	A/Victoria/55/2015(H3N2)	CY253950

**Table 2:** Molecular analysis of the deduced amino acid sequences of the A/feline/Jiangsu/HWT/2017 (H3N2) with reference strains.

	HA			NA					M2				PB2
Strains	Cleavage Site	Receptor Binding Sites		Insertion		Oseltamivir-Resistant Amino Acids			Amantadine Resistant Amino Acids				Virulence Determinant
	324–332	226	228	75–77	119	222	274	292	26	27	30	31	627
F-JS-17	PERQTRGLL	Q	G	KEI	E	I	H	R	L	V	A	S	E
F-Kor-10	PERQTRGLF	Q	G	- - -	E	I	H	R	L	V	A	S	E
F-Kor-FY-10	PERQTRGLF	Q	G	- - -	E	I	H	R	L	V	A	S	E
F-HLJ-14	PERQTRGLF	Q	G	KEI	E	I	H	R	L	V	A	S	E
F-GD-11	PEKQTRGLF	Q	G	- - -	E	I	H	R	L	V	A	S	E
F-GD-12	PEKQTRGLF	Q	G	- - -	E	I	H	R	L	V	A	N	E
C-GX-12	PEKQTRGLF	Q	G	- - -	E	I	H	R	L	V	A	S	E
D-JS-04	PEKQTRGLF	Q	G	- - -	E	I	H	R	L	V	A	S	E
Can-NJ-12	PERQTRGLF	Q	G	KEI	E	I	H	R	no	no	no	no	no
Can-GD-12	PERQTRGLF	Q	G	- - -	E	I	H	R	L	V	A	S	E
Can-HLJ-13	PERQTRGLF	Q	G	KEI	E	I	H	R	L	V	A	S	E
Can-Kor-15	PERQTRGLF	Q	G	- - -	E	I	H	R	L	I	A	S	E
Can-USA-16	PERQTRGLF	Q	G	- - -	E	I	H	R	L	I	A	S	E

F-JS-17: A/Feline/Jiangsu/HWT/2017(H3N2); F-Kor-10: A/feline/Korea/01/2010(H3N2); F-Kor-FY-10: A/feline/Korea/FY028/2010 (H3N2); F-HLJ-14: A/feline/Heilongjiang/ZH/2014; F-GD-11: A/feline/Guangdong/1/2011(H3N2); F-GD-12: A/feline/Guangdong/1/2012 (H3N2); C-GX-12:A/chicken/Guangxi/125C8/2012; D-JS-04:A/duck/Jiangsu/26/2004; Can-NJ-12:A/canine/Nanjing/11/2012; Can-GD-12:A/canine/Guangdong/12/2012; Can-HLJ-13:A/canine/Heilongjiang/L1/2013; Can-Kor-15: A/canine/Korea/S3001/2015; Can-USA-16:A/canine/Wisconsin/19137/2016. no: no sequence. - - -: no insertion.

## Data Availability

The data presented in this study are openly available in Genbank: https://www.ncbi.nlm.nih.gov/genbank/ (accessed on 30 July 2021).
